# A Case of Partial Amensia, in Which the Memory for Proper Names Was Lost

**Published:** 1834

**Authors:** 


### Art. IV.—Loss of Memory for Proper Names.

*A Case of partial Amnesia, in which the Memory for Proper Names was lost.* By the Editor.

On the 18th of January last, (1834,) a citizen of Louisville, Mr. C. Vansant, aged 45, a saddler by trade, called upon me for advice.

During the war of 1812–14, he was in service, in the neighborhood of Baltimore, and when engaged with the enemy, a musket ball passed along by his left ear and temple, so close as to graze the skin. It, in some degree, affected his senses, and gave him pain about the ear, both of which ceased after a few months. He was subsequently afflicted with long attacks of rheumatism in his limbs; but in latter years has been exempt. About 20 months ago, 18 years after the accident, he was seized, anew, with pain in the temple and around the ear of the injured side, which has continued ever since, both night and day, but is generally worse at night. Its focus is the hollow of the temple, between the ear and the eyebrow. It abates at irregular times, but returns in shooting paroxysms; and is particularly excited by locomotion, speaking, pressure on that spot, and pulling the hair near it. Perfect rest he finds most favorable to ease. In the left ear he has a constant roaring, and the meatus externus is foul; the left eye is watery; he closes it a great deal; he sometimes has pain in it: the sight of both is weak, and their motions unsteady. He has no tenderness of the spine.

About the first of the preceding September, (1833,) he had 12 "fits" (epileptic?) between 7 and 11 o'clock at night. He frothed at the mouth, and was in a state of stupor between them—which continued for two days after they ceased. Since that time, he has had two paroxysms. In regard to pain, he has been nearly in the same condition since the attack of epilepsy as before.

Every time I felt his pulse but one, it was so temperate as not to vary much from a natural condition. His bowels are generally regular. His complexion pallid, and his expression of countenance languid.

An uncommon, but not unprecedented mental effect has followed on the first attack of epilepsy. All his intellectual operations are somewhat enfeebled, and his associations now and then a little confused, but it is not these, of which I speak. *He has almost entirely lost the power of recollecting proper names, to whatever class of objects they may belong.* When he called on me, he could not tell the name of the city (Louisville) whence he came; nor that of the river, (Ohio,) nor the steam boat (Michigan) on which he had performed the voyage; nor that of the city (Cincinnati) where he then was; nor my own name. To enable himself to find me, he had written my name on a piece of paper from which he read it, when inquiring for my office. At first, (as on that visit he was alone, though his son afterwards accompanied him,) I supposed, for a moment, that he was deranged or idiotic; but soon discovered that his mind was otherwise nearly sound, for his narrative was quite intelligible and well connected, though whenever he came to a proper name, he stopped and had to substitute a description of the object, unless I myself supplied it, from the context of his discourse. I had several interviews with him, and they all presented his infirmity under the same aspect, though once or twice he succeeded, in recalling the name which was desired. In my experiments with him, I did not ask him to recollect the name of this or that object or person, but drew him into such a conversation, as involved them, and observed, that whenever he came to them, he was perplexed, displayed a countenance of anxious effort at recollecting, and seemed impatient under his inability. To give other examples of it, I may state, that he could not recollect the name of his native city, (Baltimore,) nor that of the state in which it lies, though he could converse intelligibly of both. Nor could he recall the name of the state in which he resided—that of Shelbyville, where he had once lived, nor of any of the towns around Louisville, where he then lived. He had been attended more or less, by five physicians of Louisville, one of whom was his near neighbor, but could repeat the names of none of them, though he could relate what they had done for him. Being a mechanic, he had employed journeymen, but could remember the names of none, while he could recollect and distinctly inform me of their different qualifications. In one of my interviews, he could recal the names of all his children, after studying a little; but when it came to his own baptismal name and that of his wife, he could not proceed. When he was about to leave me, on this visit, he could not ask for his umbrella, because he was unable to recal the name, which was the only instance, in our different conversations, of a failure of memory in regard to common names. Perhaps, in his mind, the idea was that of a proper more than a common noun, for at all times he used the names of common nouns, such as town, river, doctor, medicine, state, boat, &c., without the least hesitation; nor did I observe a single instance of defect of recollection of any verb, adjective, or word belonging to other parts of speech, than proper nouns. Upon putting a slate and pencil into his hands, he was *sometimes* able to write down a proper name and then read it off; but in one case he wrote "Kentucky," instead of Louisville, for the city in which he resides. In his utterance he had some degree of hesitancy, and even stammering, which led me to suppose at first that the defect was not psychological but muscular, and depended on a lesion of the facial nerve, which as Mr. Bell thinks, regulates the function of articulation—the inability, however, to write down proper names, convinced me that it was a defect of memory and not of pronunciation, though the latter was obviously somewhat impaired.

It is evident that this patient has neuralgia and epilepsy, apparently from the injury received on the left temple by the musket ball. Did that injury determine a slow morbid action of the dura mater or cranium of that part, going on to the production of exostosis, or some other organic change? Is the pain which infests that spot, of the nature of rheumatism in the periosteum? Without indulging in conjectures on these points, I shall direct the attention of the reader, to the fact, that the seat of his neuralgic pain is near to that part of the brain, which the phrenologist regards as the organ of language, situated immediately behind the globe of the eye. Without pledging ourselves to the system of organology, which has no necessary connexion with the psychological arrangement of Dr. Gall, we record this fact as deserving of preservation.

